# Insights into Osteogenesis Induced by Crude *Brassicaceae* Seeds Extracts: A Role for Glucosinolates

**DOI:** 10.3390/nu16203457

**Published:** 2024-10-12

**Authors:** Laura Gambari, Eleonora Pagnotta, Luisa Ugolini, Laura Righetti, Emanuela Amore, Brunella Grigolo, Giuseppe Filardo, Francesco Grassi

**Affiliations:** 1 Laboratorio RAMSES, IRCCS Istituto Ortopedico Rizzoli, Via di Barbiano 1/10, 40136 Bologna, Italy; laura.gambari@ior.it (L.G.); emanuela.amore@ior.it (E.A.); brunella.grigolo@ior.it (B.G.); 2 Research Centre for Cereal and Industrial Crops (CREA-CI), CREA Consiglio per la Ricerca in Agricoltura e l’Analisi dell’Economia Agraria, Via di Corticella 133, 40128 Bologna, Italy; eleonora.pagnotta@crea.gov.it (E.P.); luisa.ugolini@crea.gov.it (L.U.); laura.righetti@crea.gov.it (L.R.); 3 Faculty of Biomedical Sciences, Università della Svizzera Italiana, Viale Pietro Capelli 1, 6962 Lugano, Switzerland; giuseppe.filardo@usi.ch

**Keywords:** osteoporosis, *Brassicaceae*, *Lepidium sativum*, *Eruca sativa*, glucotropaeolin/benzylglucosinolate, glucoerucin, 4-(methylthio)butyl glucosinolate, osteogenesis

## Abstract

**Background/Objectives**: Crude extracts from the *Brassica* genus have recently emerged as promising phytochemicals for preventing bone loss. While the most documented evidence suggests that their general biological activity is due to glucosinolates’ (GLSs’) hydrolysis products, the direct activity of GLSs is beginning to be uncovered. However, the contribution of GLSs to the bone-sparing activity of crude *Brassicaceae* extracts has seldom been addressed. Here, we aimed to gain insights into this gap by studying in the same in vitro model of human osteogenesis the effect of two *Brassica* seed extracts (*Eruca sativa* and *Lepidium sativum*) obtained from defatted seed meals, comparing them to the isolated GLSs most represented in their composition, glucoerucin (GER) and glucotropaeolin (GTL), for *Eruca sativa* and *Lepidium sativum*, respectively. **Methods**: Osteogenic differentiation of human mesenchymal stromal cells (hMSCs) was assessed by alizarin red staining assay and real-time PCR, respectively, evaluating mineral apposition and mRNA expression of specific osteogenic genes. **Results**: Both *Brassica* extracts and GLSs increased the osteogenic differentiation, indicating that the stimulating effect of *Brassica* extracts can be at least partially attributed to GLSs. Moreover, these data extend previous evidence of the effect of unhydrolyzed glucoraphanin (GRA) on osteogenesis to other types of GLSs: GER and GTL. Notably, *E. sativa* extract and GTL induced higher osteogenic stimulation than *Lepidium sativum* extract and GER, respectively. **Conclusions**: Overall, this study expands the knowledge on the possible application of *Brassica*-derived bioactive molecules as natural alternatives for the prevention and treatment of bone-loss pathologies.

## 1. Introduction

Phytochemicals are plant-derived bioactive compounds that provide systemic health benefits by reducing the risk of major chronic diseases when assumed with the diet [1,2,3,4]. A growing body of evidence supports the beneficial effects of a specific dietary habit rich in phytochemicals on bone health [5,6,7,8]. For instance, adherence to the Mediterranean diet, characterized by a high intake of vegetables, fruits, legumes, and cereals, lowered the risk of hip fracture [9]; high consumption of fruits, vegetables, and seafood was associated with increased bone mineral density (BMD), delayed bone fragility, and lowered incidence of bone fractures [10,11]. Consequently, dietary recommendations have emerged as a key tool to preventing bone loss in aging and chronic diseases [7,12,13].

Among phytochemicals, organosulfur compounds (OSCs) belonging to the *Allium* and *Brassica* genera revealed broad protective activity towards organs and tissues, leading to a meaningful impact on common chronic diseases [14,15]. In particular, they are endowed with a strong potential for maintaining skeletal health [16]. Within OSCs, glucosinolates (GLSs) represent a large family of secondary plant metabolites synthesized predominantly by *Brassicaceae*, sharing the potential to form several degradation end-products when hydrolyzed by myrosinase [17]. This specific enzyme is a thioglycosidase that is well characterized in *Brassica* species and also found in bacteria, mushrooms, and insects [18,19,20]. Different chemistries of the GLSs side chains and different reaction conditions yield distinct breakdown products, with isothiocyanates (ITCs) being considered highly bioactive [21]. Importantly, chemical variations affect bioavailability and bioactivity in living organisms [22]. Even if the biological activity of GLSs is commonly attributed to the production of ITCs, direct biological activity of intact GLSs is beginning to be uncovered [23,24,25]. Our group has recently reported that glucoraphanin (GRA) obtained from Tuscan black kale exerts a beneficial effect on the osteogenic differentiation of human mesenchymal stromal cells (hMSCs; a myrosinase-free in vitro model). These data suggest that GLSs may provide a beneficial contribution to bone health independently of their conversion to ITCs [26].

Interestingly, clinical studies have demonstrated that a diet rich in food containing GLSs is associated with improved musculoskeletal health [11,27,28,29]. For example, one population-based study investigating the clinical relevance of habitual consumption of specific food for the prevention of bone fragility showed that eating *Brassicaceae* was significantly inversely associated with all fractures [11]. The use of crude extracts is essential in the preliminary screening of natural medical products [30]. Crude extracts from *Brassicaceae* showed promising results against bone-wasting diseases. The capacity to stimulate bone formation or inhibit bone resorption was reported by several authors in preclinical in vitro and in vivo studies, with reference to the effects of the ITC and to bioactive molecules from the *Lepidium* genus [16,31,32,33,34,35,36]. However, the contribution of GLSs to the bone-sparing effect of *Brassica* extracts has seldom been addressed.

Building upon these previous findings, the aim of this work was to gain further insights into the bone anabolic bioactivity of *Brassica* vegetables and the specific role of GLSs. The hypothesis was that the most prevalent GLSs may explain, at least in part, the stimulation of osteogenic activity of the native GLSs-rich extract. To test this hypothesis, we compared the activity of two *Brassica* extracts and their isolated GLSs in an in vitro myrosinase-free microenvironment during osteogenic differentiation of hMSCs. *Eruca sativa (E. sativa)* and *Lepidium sativum (L. sativum)* seeds were chosen as a source of extracts because they are rich in two types of GLSs belonging to different chemical subfamilies: glucoerucin (GER; 4-(methylthio)butylglucosinolate), an oxidized analogue of 4-methylsulfinylbutyl-GLSs (GRA), and glucotropaeolin (GTL; benzylglucosinolate), which is characterized by a benzylic side chain.

## 2. Materials and Methods

### 2.1. Extraction, Isolation, and Characterization of GLSs from Brassica Extracts

Extracts and GLSs were obtained from *E. sativa* and *L. sativum* seeds from the *Brassicaceae* collection at CREA-CI, Bologna, Italy [37]. *E. sativa* Mill. Var. NEMAT and *L. sativum* were sown in October and harvested in April–May during the 2016–2017 season within a plot with a size of 1100 m^2^, adopting a minimum agronomical input approach. Both cultivations were carried out at the CREA experimental farm located in Budrio, Bologna, in the Po Valley area (Emilia Romagna region, 44°32′00″ N; 11°29′33″ E, altitude 28 m a.s.l.). Harvesting was conducted at seed maturity when seed moisture content reached 7–8%. After harvesting, seeds were accurately cleaned and air-dried to reduce the residual moisture content and stored in a dry and dark place at room temperature until use. The ethanolic extract production included the following steps, as detailed below: seed defatting, ethanolic extraction at 80 °C of the defatted meal, and separation, concentration, and freeze-drying of the obtained extracts. *E. sativa* seeds were defatted by a small continuous seed crusher machine (Bracco Company model Elle.Gi type 0.90, Milan, Italy) with a controlled temperature procedure, as previously described [38], while *L. sativum* seeds were ground in an ultra-centrifugal mill ZM200 (Retsch GMBH, Haan, Germany), sieved at 0.75 mm, and defatted using hexane (1:10 *w*/*v*) under agitation overnight at room temperature. The solvent de-oiling in the case of *L. sativum* was necessary to more effectively remove the particularly abundant mucilages from its seeds during extraction procedures. Both *E. sativa* and *L. sativum* defatted seed meals were ground to 500 µm grain size. A total of 300 g of homogeneous meals were extracted in 3 L of 70% ethanol for 10 min at 80 °C with an Ultra-Turrax homogenizer (IKA-Werk, Staufen, Germany). The extracts were cooled in an ice bath and then centrifuged at 25,900× *g* for 30 min at 10 °C. The supernatants were reduced about three-fold at 40 °C in rotavapor, stored at −20 °C for 48 h, and finally freeze-dried (DLAB 500, Italian Vacuum Technology, Trezzano sul Naviglio, Italy). *E. sativa* and *L. sativum* powdered extract were analyzed for GLSs content by means of HPLC-UV of previously desulfated GLSs, according to procedures previously described in Testai et al. [39]. Briefly, desulfation was achieved by loading extracts and purified sulfatase (0.35 U mL-1, 200 μL, and 100 μL for *E. sativa* and *L. sativum* extracts) onto a mini column filled with DEAE Sephadex A-25 anion-exchange resin (Cytiva, Uppsala, Sweden). The desulfated GLSs were eluted in water and detected by monitoring their absorbance at 229 nm by using a Hewlett-Packard 1100 HPLC equipped with a diode array detector and an Inertsil 5 ODS-3 column (250 × 3 mm, 5 µm). The desulfo-GLSs were identified by UV spectra and HPLC retention times according to a purified standard library. The amount was estimated through the internal standard method (by using purified sinigrin as the internal standard) and published response factors [40].

Purified GER from *E. sativa* defatted seed meals and GTL from *L. sativum* seeds were isolated according to the chromatographic two-step procedure described in Flori et al. [41]. Individual fractions containing pure GLSs in distilled water eluted from the final step of purification by gel filtration chromatography were pooled and freeze-dried before HPLC-UV analysis, which was performed as detailed above.

### 2.2. Cell Isolation and Culture

The study was approved by the Institutional Ethic Committee (CE 0011810/2017) and conducted according to the principles of the ICH-GCP. All surgical procedures and the harvesting of human tissues were performed at the Rizzoli Orthopedic Hospital after obtaining the patients’ informed consent. hMSCs were isolated by using a mechanical and a ficoll-density gradient isolation protocol (Cederlane, Burlington, ON, Canada) from bone chips of tibial plateau obtained by patients undergoing surgical knee replacement [42]. Cells were cultured in α-MEM 15% FBS (Thermofisher Waltham, US; Euroclone, Pero, Italy) at 37 °C and in 5% CO_2_ and 95% O_2_, and medium was replaced twice per week; they were expanded until passage 2, harvested with trypsin/EDTA solution 0.25% (Biochrom, Berlin, Germany), and seeded for further experiments. A total of 8 donors of hMSCs (mean age 67 ± 10, BMI 25 ± 3) were employed to further assess the osteogenic differentiation (after culture with osteogenic factors in the presence or absence of extracts or purified GLSs) by a functional evaluation of bone mineral matrix production (by alizarin red staining) and by a quantitative evaluation of mRNA expression of osteogenic markers (by real-time PCR).

### 2.3. Osteogenic Differentiation and Alizarin Red Staining

Each analysis was performed in duplicate. hMSCs were seeded at 5 × 10^4^ cells/cm^2^ in a 12-well plate in α-MEM 15% FBS and cultured for 14–21 days in osteogenic medium (α-MEM 20% FBS supplemented with 100 nM dexamethasone, 100 µM ascorbic acid, and 10 mM β-glycerophosphate; Thermofisher, Waltham, MA, USA; Euroclone, Milan, Italy; Sigma Aldrich, St. Louis, MO, USA) with or without treatment with purified GLSs at increasing doses (10–30–100 μM) or extracts at the same concentration expressed in GLSs content (GER and GTL for *E. sativa* and *L. sativum* extract, respectively). Medium and stimuli were replaced twice per week. At D14 and D21, Alizarin Red S (AR-S) (Sigma Aldrich,) staining was performed to assess the presence and extent of mineralization. Briefly, cells were stained with 40 mM AR-S for 20 min, after being fixed for 15 min at RT in formaldehyde (Kaltek, Padova, Italy) 10% phosphate-buffered saline (PBS, Thermofisher) and washed twice with PBS, as detailed elsewhere. Cell positivity at AR-S staining was observed with a Nikon Instruments Europe BV optic microscope (Amstelveen, The Netherlands), and pictures were taken at 100× of magnifications.

### 2.4. AR-S Staining Quantification and Evaluation

A spectrophotometric analysis at 510 nm with TECAN Infinite^®^ 200 PRO (Tecan Italia S.r.l., Cernusco Sul Naviglio, Italy), recording 177 readings for each well (in duplicate), was performed to quantify the mineral apposition, as described in our previous studies [26,42,43]. First, the 177 readings obtained from all 8 donor hMSCs treated with each stimulus (in duplicate) were averaged to have a comprehensive overview of the effect of each treatment. Then, based on the renowned heterogeneous ability of differentiation of different hMSCs donors, we analyzed whether this aspect could be a possible confounding factor. Therefore, to gain further insights into the effect of each treatment limiting this possible confounding effect, we analyzed each donor for its ability to induce mineral apposition at D14 and D21. Particularly, we considered 0.3 as the threshold value corresponding to the baseline mineralization and 0.9 as the threshold value corresponding to high levels of mineralization [26,42,43]. A detection (1) below the threshold of 0.3 identified cells that were not producing mineral matrix, (2) between 0.3 and 0.9 identified cells that were producing low mineral matrix, and (3) above 0.9 identified cells that were producing high mineral matrix. Based on the above considerations, we evaluated the effect of each treatment comparing (a) all the donors, (b) the non-mineralizing donors (in analysis at D14) or low-mineralizing donors (in analysis at D21) and (c) the high-mineralizing donors.

### 2.5. Quantification of mRNA Expression by Real-Time PCR

hMSCs were cultured under osteogenic stimulation (as detailed above) to assess the mRNA expression of some of the most relevant genes involved in osteogenesis: alkaline phosphatase (ALP), bone sialoprotein (BSP), SMAD Family Member 1 (SMAD-1), and WNT1-inducible-signaling pathway protein 1 (WNT-1). At the end of the culture, duplicates of cells were lysed using 1 mL of pure RNA solution (Euroclone) before performing the chloroform–phenol–ethanol (Invitrogen, Waltham, MA, USA) extraction protocol and the purification from genomic DNA by treatment with DNase I (DNA-free Kit, Ambion, Austin, TX, USA), according to the manufacturer’s instructions. cDNA synthesis was performed by using a SuperScript™ VILO™ cDNA Synthesis Kit (Invitrogen) on a 2720 Thermal Cycler (Applied Biosystem, Life Technologies, Waltham, MA, USA) at 25 °C for 10 min, 42 °C for 60 min, 85 °C for 5 min, and 4 °C for 30 min. mRNA expression was assessed by real-time polymerase chain reaction (PCR) analysis using the SYBR Premix Ex Taq (TaKaRa Biomedicals, Tokyo, Japan). Primers were purchased from Life Technologies Italia and are listed in Table 1. The real-time PCR analyses were run on a LightCycler Instrument (Roche, Basel, Switzerland) as follows: one cycle at 95 °C for 10 s, at 45 cycles at 60 °C for 20 s, and at 95 °C for 5 s. Standard melting curve analyses were performed at 95 °C for 10 s, 65 °C for 15 s, and 95 °C reached in one-degree increments to confirm the specificity of the PCR products. The PCR products were relatively quantified with the comparative CT method, normalizing to the housekeeping mRNA expression of glyceraldehyde-3 phosphate dehydrogenase (GAPDH).

### 2.6. Statistical Analyses

GraphPad Prism 7 (La Jolla, CA, USA) was used for statistical analyses. Before each test, the presence of outliers was checked by a ROUT (Q = 1%) test. Outliers were removed from each data set when present. The D’Agostino and Pearson normality test was performed to analyze the normality of the data. We performed a Friedman + Dunn multiple-comparisons test to analyze AR-S data, a “two-way ANOVA” to evidence the effect of both concentration and donor effect independently on AR-S quantification evaluations, and a two-way ANOVA and Tukey’s multiple comparisons test for analysis of gene expression. Significance was attributed when *p* < 0.05 (*), *p* < 0,01 (**), *p* < 0.001 (***), *p* < 0.0001 (****).

## 3. Results

### 3.1. Characterization of Extracts and Purified GLSs

**Figure 1:**
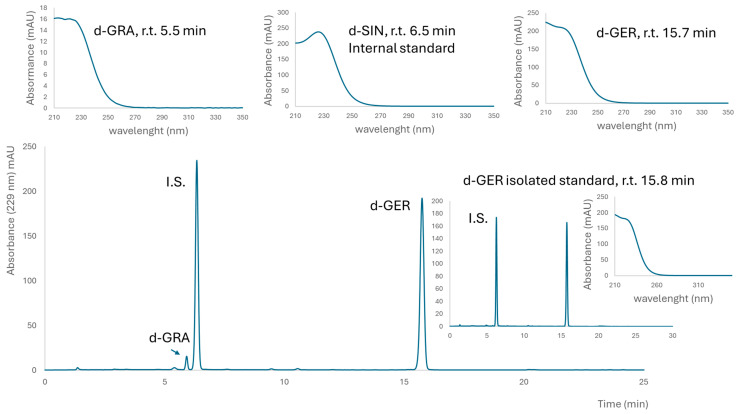
**Typical chromatogram of desulfated glucosinolates in the *E. sativa* extract.** Above UV spectra of internal standard (I.S.) desulfo sinigrin (SIN) and identified desulfoglucosinolates, glucoraphanin (GRA) and glucoerucin (GER) are reported with their retention times (r.t.). The insert on the right shows a chromatogram of purified GER and I.S. for the determination of purity on a weight basis.

**Figure 2:**
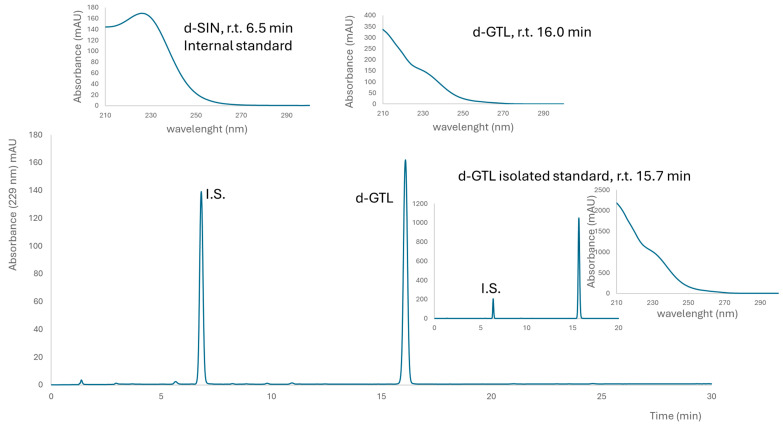
**Typical chromatogram of desulfated glucosinolates in the *L. sativum* extract.** Above UV spectra of internal standard (I.S.) desulfo sinigrin (SIN) and glucotropeaolin (GTL) are reported with their retention times (r.t.). The insert on the right shows a chromatogram of isolated GTL and I.S. for the determination of purity on a weight basis.

A brown powder containing 498 ± 6 μmol g^−1^ GER and 22 ± 2 μmol g^−1^ GRA was obtained from *E. sativa* seeds. This lyophilized extract contained 22% (*w*/*w*) total GLSs, with a preponderant amount of GER, which was 21% (*w*/*w*). A fine yellow powder containing 752 ± 40 μmol g^−1^ GTL (as the only detectable GLSs) was obtained from *L. sativum* seeds. This lyophilized extract contained 31% (*w*/*w*) GTL. Purified GER and GTL were isolated as K+ salts from *E. sativa* and *L. sativum* defatted seed meals, respectively, and their purity, assessed by HPLC-UV, was 99% (area peak based) and 97% on a weight basis for GER, and 99% as indicated both by HPLC-UV and on a weight basis for GTL (Figure 1 and Figure 2).

### 3.2. Brassica-Derived Extracts and GLSs Stimulate Mineral Deposition

To identify the key component of the extracts’ biological activity, we performed a matched analysis of the effect of GLSs-rich extracts and of their purified GLSs in a myrosinase-free environment: in vitro cultures of hMSCs (harvested from 8 different donors) treated with osteogenic stimulation with or without purified GLSs (GER or GTL) or crude extracts (*E. sativa* and *L. sativum*).

**Figure 3:**
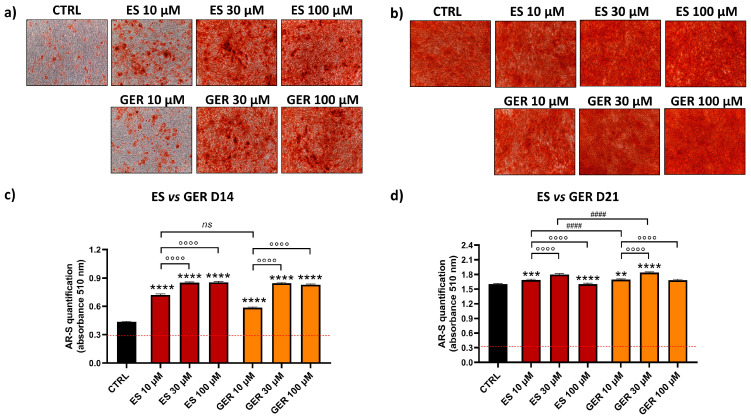
**AR-S on ES- and GER-treated cells vs. CTRL cells during osteogenic stimulation.** Panels (**a**,**b**) show representative images at D14 ARS (**a**) and D21 (**b**). Panels c and d show histograms (mean ± SEM) of AR-S quantification obtained by 177 measurements in duplicate for each of the N = 8 donors comparing ES (red), GER (orange), CTRL (black) at D14 (**c**) and D21 (**d**). Friedman–Dunn’s multiple-comparisons test: *: Comparisons between CTRL and treatments (at each concentration); °: Comparisons between different concentrations of each treatment (10 μM vs. 30 μM; 10 μM vs. 100 μM); #: Comparisons between different treatments at the same concentration (es 10 μM ES vs. 10 μM GER). ** *p* < 0.01; *** *p* < 0.001; ****, °°°°, #### *p* < 0.0001, *ns* = non significant. ES extract concentration is expressed as GER content (μM).

**Figure 4:**
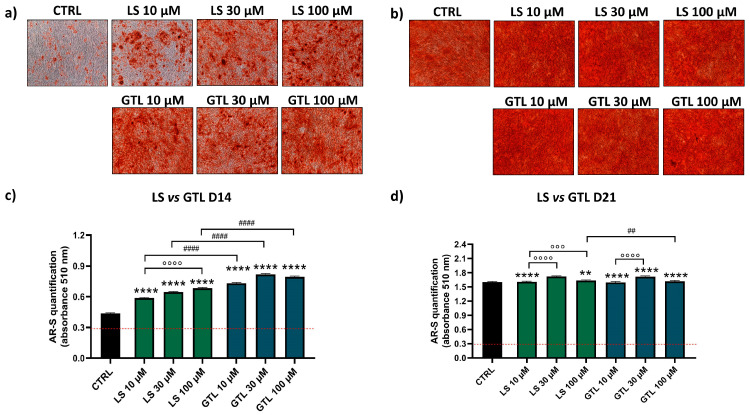
**AR-S on LS- and GTL-treated cells vs. CTRL cells during osteogenic stimulation.** Panels a and b shows representative images at D14 ARS (**a**) and D21 (**b**). Panels (**c**,**d**) show histograms (mean ± SEM) of AR-S quantification obtained by 177 measurements in duplicate for each of the N = 8 donors comparing LS (green), GTL (blue), CTRL (black) at D14 (**c**) and D21 (**d**). Friedman–Dunn’s multiple-comparisons test: *: Comparisons between CTRL and treatments (at each concentration); °: Comparisons between different concentrations of each treatment (10 μM vs. 30 μM; 10 μM vs. 100 μM); #: Comparisons between different treatments at the same concentration (es 10 μM ES vs. 10 μM GER). **, ## *p* < 0.01; °°° *p* < 0.001; ****, °°°°, #### *p* < 0.0001. LS extract concentration is expressed as GTL content (μM).

AR-S was performed to detect the amount of mineral deposition in the presence or absence of phytochemical stimulation. Representative images of one donor show cell monolayers with different levels of red positivity in cells treated only with osteogenic stimulation (CTRL) or treated with extracts/GLSs at D14 (Figure 3a,b and Figure 4a,b). The quantification of AR-S by spectrophotometric measurements in the eight donors analyzed showed a high stimulation of differentiation both for GLSs-rich extracts and isolated GLSs as compared to CTRL at D14 (Figure 3c and Figure 4c).

When compared to CTRL, *E. sativa* (ES) extract increased the amount of mineral deposition by 1.7-fold (10 μM ES), 1.9-fold (30 μM ES), and 2-fold (100 μM ES) at D14 (Figure 3c; **** *p* < 0.0001). Moreover, low doses of ES (10 μM) showed lower and significant values compared to greater doses (30–100 μM), showing a dose-dependent effect of this treatment (°°°° *p* < 0.0001). Glucoerucin (GER), the main GLSs constituent of ES, showed a similar stimulatory effect on osteogenesis. When compared to CTRL, GER increased the amount of mineral deposition by 1.4-fold (10 μM GER), 1.9-fold (30 μM GER), and 1.9-fold (100 μM GER) at D14 (Figure 3c; **** *p* < 0.0001), showing a dose-dependent effect (°°°° *p* < 0.0001). At D21, when most donors reached the highest level of matrix production, a negligible additive effect on osteogenic stimulation was found either for ES or for GER. When the ES effect was directly compared to the GER effect, no difference was found at D14 (Figure 3d); a low and significant increase was found at D21 for GER 10 and 30 μM (#### *p* < 0.0001).

Similarly, *Lepidium Sativum* (LS) extract and its most representative GLSs constituent, GTL, showed a positive effect on osteogenic stimulation. Compared to control samples, LS increased mineral deposition by 1.3-fold (10 μM LS), 1.5-fold (30 μM LS), and 1.6-fold (100 μM LS) at D14 (Figure 4c; **** *p* < 0.0001), showing a dose-dependent effect (°°°° *p* < 0.0001). Isolated GTL was able to induce osteogenesis with higher amounts compared to LS (#### *p* < 0.0001; at 10-30-100 μM, D14). GTL increased the amount of mineral deposition by 1.7-fold (10 μM GTL), 1.9-fold (30 μM GTL), and 1.8-fold (100 μM GTL) at D14 (Figure 4c; **** *p* < 0.0001), showing no dose-dependent effect. At D21, when the level of mineralization almost reached the plateau, only a negligible increase in mineral apposition was found for both LS and GTL (Figure 4d).

**Figure 5:**
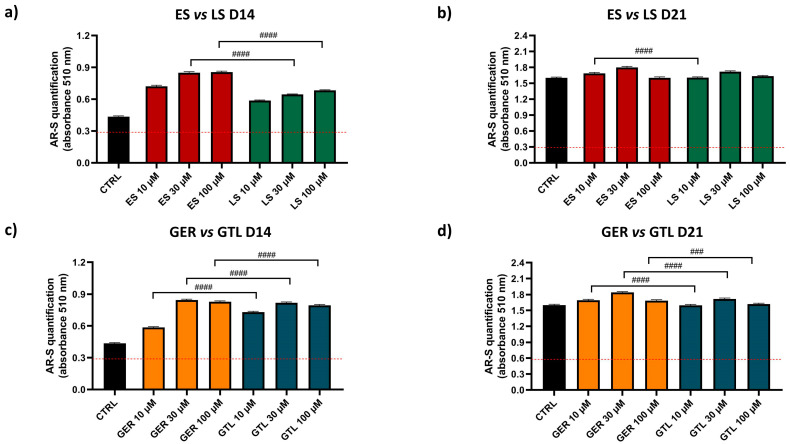
AR-S on ES-, GER-, LS-, and GTL-treated cells vs. CTRL cells during osteogenic stimulation. Panels (**a**–**d**) show histograms (mean ± SEM) of AR-S quantification obtained by 177 measurements in duplicate for each of the N = 8 donors comparing (**a**) ES (red) vs. LS (green) D14; (**b**) ES vs. LS D21; (**c**) GER (orange) vs. GTL (blue) (D14); (**d**) GER vs. GTL (D21). Friedman–Dunn’s multiple-comparisons test: #: Comparisons between different treatments at the same concentration (es 10 μM ES vs. 10 μM GER). #### *p* < 0.0001. ES and LS extract concentrations are expressed as GER and GTL content (μM), respectively.

Further statistical analysis directly compared the extracts in order to provide more information on whether ES or LS displays more osteogenic properties in the same model (as shown in Figure 5). ES showed higher stimulation compared to LS at concentrations of 30 and 100 μM (#### *p* < 0.0001) at D14 and at a concentration of 10 μM (#### *p* < 0.0001) at D21. Similarly, we directly compared purified GLSs to provide more information of whether GER or GTL displays more osteogenic properties in the same model. GTL showed higher stimulation compared to GER at each concentration tested at D14 (# *p* < 0.0001), while GTL at D21 showed negligibly lower values compared to GER.

To gain further insights into the variables affecting this response, we performed a “two-way ANOVA” considering concentrations of stimulus and the donor as possible sources of variation. As summarized in Table 2, at both time points the cell donor turned out to be a significant source of variation.

### 3.3. Brassica-Derived Extracts and GLSs Stimulate Greater Amounts of Mineral Deposition in the Non-/Low-Mineralizing Subgroups of Donors

**Figure 6:**
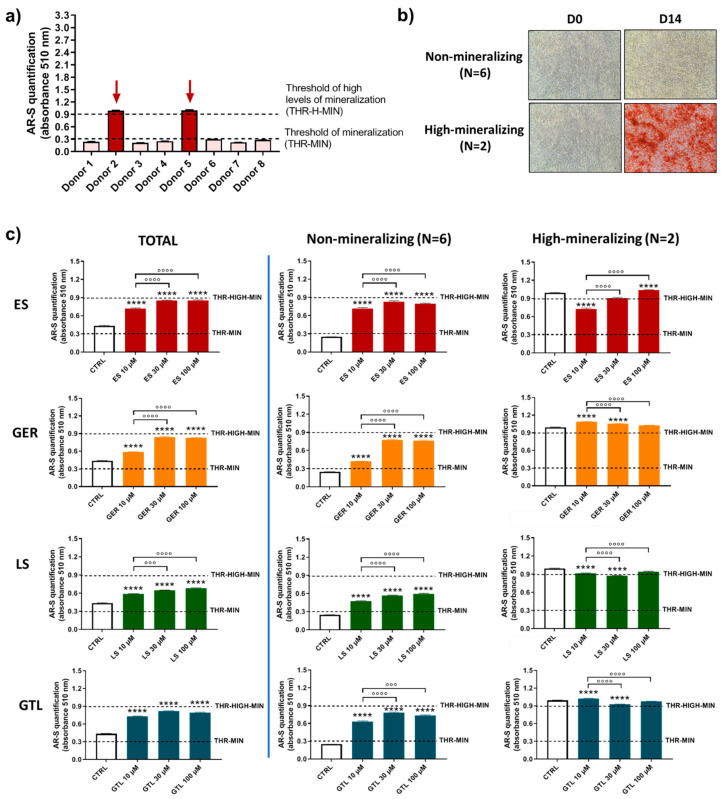
**AR-S on ES-, GER-, LS-, and GTL-treated cells vs. CTRL cells during osteogenic stimulation in the total population and selected groups of donors at D14**. (**a**) Histograms (mean ± SEM) of AR-S quantification of 177 values in duplicate for each one of 8 donors, highlighting donors displaying different levels of mineral deposition. Arrows indicate donors displaying early high levels of mineral matrix deposition. (**b**) Representative images of AR-S at D0 and D14 (magnification 10×), highlighting the two subgroups of donors found at D14: non-producing mineral matrix (non-mineralizing) and highly producing mineral matrix (high-mineralizing). (**c**) Histograms (mean ± SEM) of AR-S of 177 values in duplicate for each one of 8 donors (TOTAL), non-mineralizing donors (N = 6) and high-mineralizing donors (N = 2) for ES (red), GER (orange), GTL (blue), and LS (green). Friedman–Dunn’s multiple-comparisons test: *: Comparisons between CTRL and treatments (at each concentration); °: Comparisons between different concentrations of each treatment (10 μM vs. 30 μM; 10 μM vs. 100 μM). °°° *p* < 0.001; ****, °°°° *p* < 0.0001. ES and LS extract concentrations are expressed as GER and GTL content (μM), respectively.

**Figure 7:**
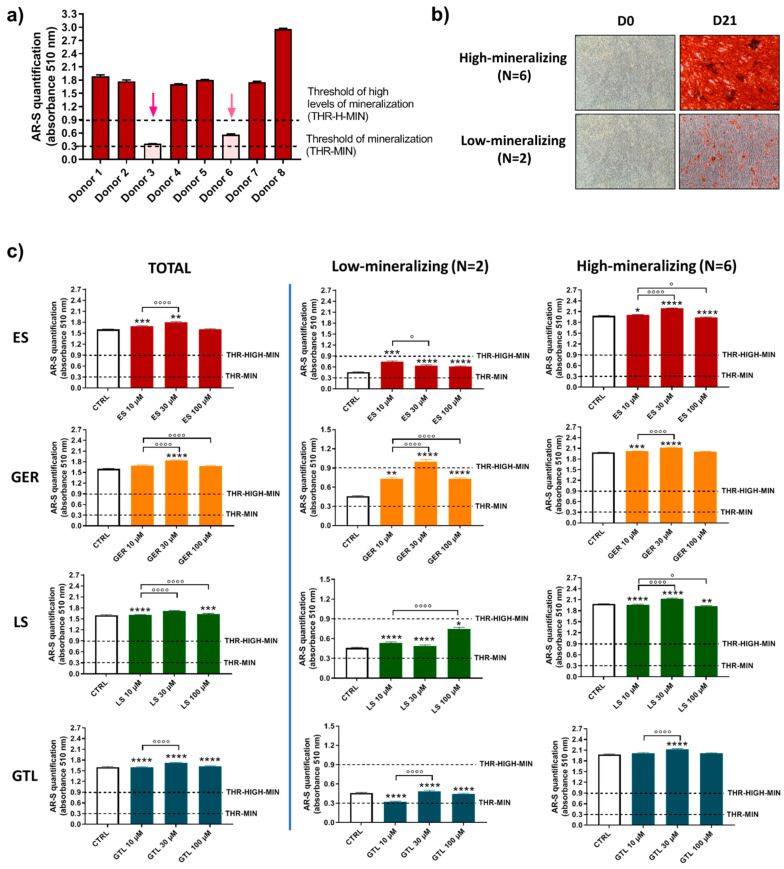
**AR-S on ES-, GER-, LS-, and GTL-treated cells vs. CTRL cells during osteogenic stimulation in the total population and selected groups of donors at D21.** (**a**) Histograms (mean ± SEM) of AR-S quantification of 177 values in duplicate for each of 8 donors, highlighting donors displaying different levels of mineral deposition. Arrows indicate donors displaying low levels of mineral matrix deposition. (**b**) Representative images of AR-S at D0 and D21 (magnification 10×), highlighting the two subgroups of donors found at D21: low-producing mineral matrix (low-mineralizing) and highly producing mineral matrix (high-mineralizing). (**c**) Histograms (mean ± SEM) of AR-S of 177 values in duplicate for each of 8 donors (TOTAL), low-mineralizing donors (N = 2) and high-mineralizing donors (N = 6) for ES, GER, GTL, and LS. Friedman–Dunn’s multiple-comparisons test: *: Comparisons between CTRL and treatments (at each concentration); °: Comparisons between different concentrations of each treatment (10 μM vs. 30 μM; 10 μM vs. 100 μM). *, ° *p* < 0.05, ** *p* < 0.01, ***, ****, °°°°, *p* < 0.0001. ES and LS extract concentrations are expressed as GER and GTL content (μM), respectively.

It was previously described that primary hMSCs subjected to osteogenic differentiation media show different mineralizing behavior [42]. Based on this previous knowledge and the evidence that donors influence hMSCs’ mineralizing response to *Brassica* seed extracts/GLSs stimulus; we analyzed more in detail the population of eight different donors of hMSCs and further evaluated the effects of GLSs and GLSs-enriched extracts in different subgroups. By analyzing the levels of mineral matrix produced by cells, we identified different “subgroups” at D14 and D21. At D14 (Figure 6a,b), the majority of donors were not-mineralizing at this stage (values below the threshold of mineralization; N = 6), while only two donors displayed high levels of mineralization (values around 0.9; N = 2). At D21 (Figure 7a,b), the majority of donors (N = 6) were highly mineralizing at this stage (values above the “threshold of high levels of mineralization”, ranging from 1.74 to 2.96), while two cases displayed low values of mineralization (0.36 and 0.56; Figure 4c).

Figure 6c shows a comparison among (1) the entire population (TOTAL), (2) the group of cells not producing mineral matrix at D14 (non-mineralizing; N = 6), and (3) the group of donors producing early high levels of mineral matrix at D14 (high-mineralizing; N = 2). When we analyzed AR-S quantification, in the non-mineralizing donors (N = 6) we found the highest ability of stimulation for all compounds tested compared to CTRL cells. ES increased the amount of mineral deposition by 2.9-fold (10 μM ES), 3.2-fold (30 μM ES), and 3.2-fold (100 μM ES); GER increased the amount of mineral deposition by 1.7-fold (10 μM GER), 3.0-fold (30 μM GER), and 3.0-fold (100 μM GER). LS increased the amount of mineral deposition by 1.9-fold (10 μM LS), 2.3-fold (30 μM LS), and 2.4-fold (100 μM LS); GTL increased the amount of mineral deposition by 2.5-fold (10 μM GTL), 3.1-fold (30 μM GTL), and 2.9-fold (100 μM GTL) D14. GER. When we analyzed AR-S quantification, in the group producing early high levels of mineral matrix (high-mineralizing) (N = 2), we found only negligible differences for all compounds tested compared to CTRL cells, thus evidencing that the stimulus of *Brassica* seed extracts and both isolated GLSs is ineffective when the mineralization is highly advanced.

Confirming this interpretation, there were only negligible differences in the entire population and in the high-mineralizing subgroup at D21 (Figure 7c). On the other hand, when we analyzed AR-S quantification, selecting the two cases that displayed low levels of mineralization at D21, we still found an ability to stimulate the osteogenesis for most compounds tested. In particular, ES increased the amount of mineral deposition by 1.6-fold (10 μM ES), 1.4-fold (30 μM ES), and 1.3-fold (100 μM ES); GER increased the amount of mineral deposition by 1.6-fold (10 μM GER), 2.2-fold (30 μM GER), and 1.6-fold (100 μM GER). Similarly, LS increased the amount of mineral deposition by 1.2-fold (10 μM LS), 1.1-fold (30 μM LS), and 1.6-fold (100 μM LS), while unlike ES, LS, and GER, no stimulatory effect was found for GTL. 

Overall, these data confirm that donors are a source of variation when analyzing the effect of both extracts and GLSs on mineral deposition, although this aspect seems to be strictly dependent on the level of mineralization of the CTRL cells. In particular, we found that, independent of the time of evaluation, a high increase in mineral matrix deposition by GLSs/extracts was found only in the low-/non-mineralizing group of donors, while only minor variations were found in the high-mineralizing group.

### 3.4. Brassica-Derived Extracts and GLSs Stimulate the Expression of Osteogenic Markers in hMSCs

To date, only a few studies have investigated the modulation of osteogenic genes or osteogenic protein by GLSs in MSCs or osteoblasts, showing increased collagen protein synthesis, increased ALP activity, and increased BSP and SMAD-1 mRNA expression [26,35].

To gain further insights into the molecular signaling involved in the regulation of osteoprogenitor cells, we also evaluated the gene expression of several signature markers of osteogenic differentiation. In particular, SMAD-1 is involved in TGF-β and BMP signaling and promotes osteoblast differentiation and proliferation [44]; WISP1 regulates bone remodeling during aging by modulating osteoblast and, in particular, Wnt signaling [45]; BSP is abundantly expressed in mineralized tissues, where it plays a role in matrix mineralization [46]; and ALP plays a critical function in the formation of mineralized tissue [47].

Given the above-mentioned differences in mineralization processes, we restricted the analysis to the most homogeneous groups: (1) non-mineralizing donors at D14 and (2) high-mineralizing donors at D21.

**Figure 8:**
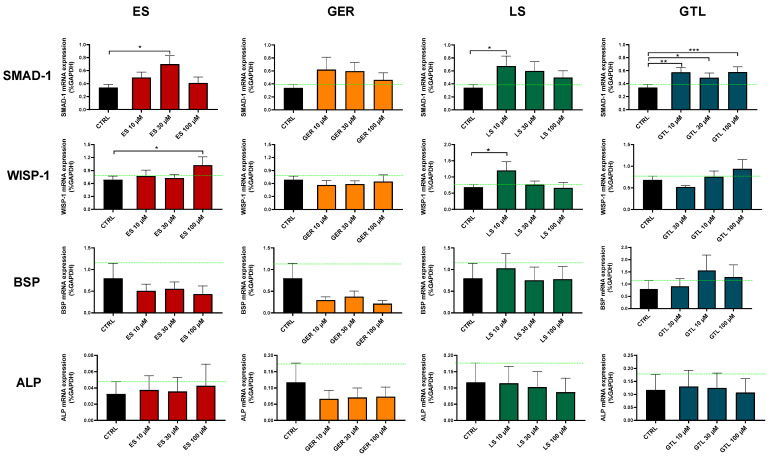
mRNA expression of osteogenic markers in ES-, GER-, LS-, and GTL-treated cells vs. CTRL cells during osteogenic stimulation at D14. Histograms show mean ± SEM of mRNA expression in duplicate for each of 6 donors (for SMAD-1, WISP-1, BSP, and ALP) for ES (red), GER (orange), LS (green), GTL (blue). Two-way ANOVA and Tukey’s multiple-comparisons test were performed. * *p* < 0.05, ** *p* < 0.01, *** *p* < 0.001.

**Figure 9:**
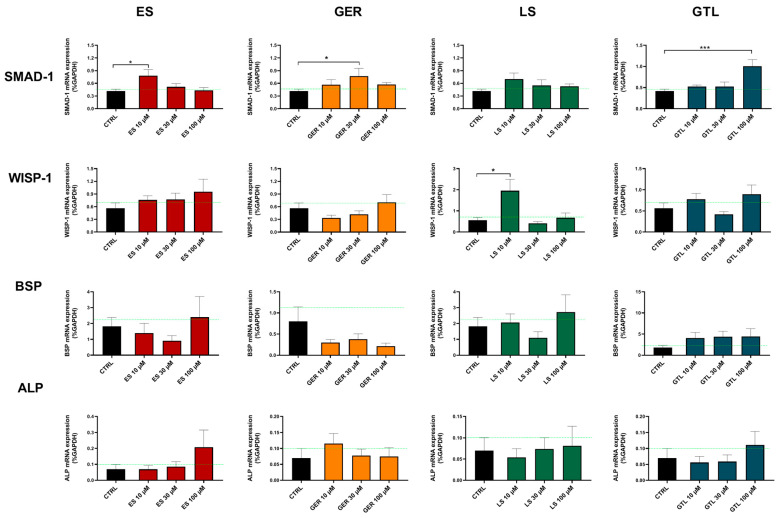
mRNA expression of osteogenic markers in ES-, GER-, LS-, and GTL-treated cells vs. CTRL cells during osteogenic stimulation at D21. Histograms show mean ± SEM of mRNA expression in duplicate for each of 6 donors (for SMAD-1, WISP-1, BSP, and ALP) for ES (red), GER (orange), LS (green), GTL (blue). Two-way ANOVA and Tukey’s multiple-comparisons test were performed. * *p* < 0.05, *** *p* < 0.001.

Among the markers tested here, SMAD-1 was the one most regulated at both D14 and D21 (Figure 8 and Figure 9). At D14, GTL activated SMAD-1 expression at each concentration tested: 10 μM (*p* < 0.01), 30 μM (*p* < 0.05), and 100 μM (*p* < 0.001), while ES at 30 μM (*p* < 0.05), LS at 10 μM (*p* < 0.05), and GER displayed a non-significant increasing trend (Figure 8). At D21, only GTL at 100 μM (*p* < 0.001), ES at 10 μM (*p* < 0.05), and GER at 30 μM (*p* < 0.05) showed significant stimulation of expression (Figure 9). WISP-1 was significantly up-regulated by ES at 100 μM (*p* < 0.05) at D14 (Figure 8) and LS at 10 μM (*p* < 0.05) at D14 and D21 (Figure 8 and Figure 9). Only GER at 100 μM (*p* < 0.05) significantly up-regulated BSP expression at D21 (Figure 9). Neither extracts nor GLSs further increased ALP expression with respect to CTRL cells (Figure 8 and Figure 9).

Overall, SMAD-1 was the only marker up-regulated by most of the phytochemicals. Particularly, GTL was the only phytochemical inducing significant stimulation at each concentration tested at D14 (Figure 8). Therefore, we analyzed its expression in different subgroups of donors based on different mineralization status and we found that GTL differently induced SMAD-1 if analyzed in the donors not yet producing mineral matrix (where it was up-regulated, *p* < 0.05) and in the ones producing high levels of mineral matrix (where it was not up-regulated), which is coherent with the mineralization data (Supplementary Figure S1).

## 4. Discussion

Osteoporosis is a subtle condition that is still underestimated in the population and often diagnosed only when fragile bone fracture occurs. Phyto-compounds and nutraceuticals are attracting particular interest in this context, given their promising perspectives in the prevention of bone loss and fracture. Plants belonging to the *Brassicaceae* family are the main source of natural GLSs, which are sulfur-containing molecules that are part of the defense system of these vegetables against pathogens. Several reports have shown that extracts obtained from different parts of these plants may effectively stimulate osteoblast activity and slow the progression of bone erosion in in vitro and in vivo models. However, *Brassica* extracts that have been tested are limited to *Brassica rapa L. root*, *Lepidium meyenii Walp. Brassica oleracea*, and, to a larger extent, *Lepidium sativum*. Moreover, despite the interest in studying crude extracts as a source of medical bioactive natural products, for *Brassicaceae* no studies have performed a bio-fractionation approach to correlate the effect of crude extracts to that of specific bioactive molecules of interest purified from them.

A previous study from our group using a myrosinase-free in vitro model showed that GLSs are not biologically inert molecules, given that GRA, a GLSs found in broccoli and turnip, was entailed with osteogenic properties even in the absence of its derived ITC, sulphoraphane [26]. To investigate whether other classes of GLSs have the same biological properties in human bone cells, we extended our investigation to different chemical subgroups of GLSs. These share a common core structure consisting of an S-β-D-glucopirano unit linked to an O-sulfated-(Z)-thiohydroximate and a variable side chain. Here, we studied GER, which represents an oxidized analogue of 4-methylsulfinylbutyl-GLSs, GRA, and has been identified as responsible, at least in part, for the beneficial effects of *E. sativa* seeds on the cardiovascular system [48], on intestinal inflammation and pain and on diabetic neuropathy [38,48]. Moreover, we studied GTL, an aromatic hydrophylic compound present in several plants with known anti-inflammatory and antioxidant effects [49,50]. Like GRA, when hMSCs were stimulated with different concentrations of GER and GTL (10, 30, and 100 μM), a marked increase in the mineral matrix apposition was observed, especially at D14. In our previous work, we found that GRA stimulation mostly increased the expression of BSP and SMAD1 while inducing occasional down-regulations of ALP and WISP1. Here, BSP was increased only by GER, while SMAD-1 was up-regulated by all the phytochemicals analyzed and especially by GTL at D14. Taken together, these data suggest that the mechanism beyond osteogenic induction is common to different groups of GLSs and independent of the chemical structure.

To gain more insights into the possible contribution of GLSs in the effect of crude extracts, we studied the osteogenic stimulation of crude extracts containing GER and GTL, from *E. sativa* and *L. sativum*, respectively. Notably, our data indicate that both extracts induce osteogenesis similarly to their most prevalent GLSs. Because we used the same human cellular model, we can conclude that *E. sativa* and *L. sativum* extracts’ stimulatory effect can be at least partially attributed to GER and GTL.

Notably, while several studies previously investigated the effects of *L. sativum* extracts in preclinical models of bone loss [16], our findings represent the first preliminary evidence of the efficacy of *L. sativum* extracts on human bone cells. In vivo studies in rodents demonstrated the ability of *L. sativum* extracts to prevent osteoporosis by modulating bone remodeling [31,33,34] and to improve fracture healing [32,36]. In particular, *L. sativum* extracts increased markers of bone formation (ALP, OCN) and decreased markers of bone resorption (TRAP, CTX-I) in serum [31,34], increased femur strength [31], increased the percentage of trabecular bone [34], and increased cortical thickness in femur, osteoblast, OPN expression, and RANKL/OPG ratio [31], while it decreased the number of osteoclasts [33]. Seeds, leaves, roots, and seed oil of *L. sativum* are a rich source of alkaloids, GLSs, saponins, terpenes, and saturated and essential fatty acids. The specific phytocompounds responsible for its action are still unknown and worthy of investigation. 

Notably, here, we studied the effect of *L. sativum* seed extracts and GTL on hMSCs derived from the same donors. GTL is the only detected GLSs in the *L. sativum* seed extracts, representing 31% of the total composition. Our data show that GTL increased the mineralization more than *L. sativum* extract, evidencing that the effect of *L. sativum* extract is at least partially attributed to the effect of GTL. Moreover, we can exclude an effect of ITCs (such as BITCs) and of saturated or essential fatty acids since this extract was produced by the inactivation of myrosinase at high temperature and was produced starting from defatted seed meal. Further studies should be directed to investigating the specific role of other compounds in *L. sativum*.

Unlike *L. sativum* extract, neither *E. sativa* extract nor *E. sativa*-derived GLSs stimulating osteogenesis has ever been investigated in the literature. Interestingly, we found that *E. sativa* extract is able to increase osteogenesis on hMSCs; moreover, a direct comparison revealed that *E. sativa* extract induced higher osteogenic stimulation than *L. sativum* extract. In particular, we found that GLSs comprised 22% (*w*/*w*) of the *E. sativa* extract and that GER and GRA represented 21% and 1% of GLSs composition, respectively. Here, we tested GER in the same model of osteogenesis, and we found that it induced osteogenesis compared to CTRL hMSCs, thus highlighting that *E. sativa* extract’s effect can be at least partially attributed to the effect of GER. Notably, we found higher osteogenic ability for *E. sativa* extract than for GER. We previously showed that GRA induces the osteogenic differentiation of hMSCs; thus, we can speculate on the synergic activity of GER and GRA or the additive–synergic action of other compounds. Notably, the *E. sativa* seed extracts used were depleted of fatty acid and were composed, along with GLSs, of phenol and flavonoids. Overall, these data are the first evidence reporting increased osteogenesis by both *E. sativa* seed extracts and GER.

Another interesting aspect of the study findings is the different response to both extracts and GLSs in hMSCs grouped according to different mineralizing behavior during osteogenic differentiation. We previously showed that primary hMSCs display a different response to osteogenic stimuli and can be grouped in low- or high-mineralizing cells depending on the amount of mineralization laid on the monolayer during a 4-week osteogenic culture [42]. Here, we showed that both crude extracts and GLSs induce a stronger response in low-mineralizing MSCs, even if at different extents. While the mechanism underlying this phenomenon is not clear, it is tempting to speculate that GLSs may supply exogenous hydrogen sulfide (H_2_S), an important stimulus for osteogenic differentiation [51]. Recently, the interest in categories of plant foods that are a natural source of H_2_S has risen and their relevance in bone tissue has emerged. In particular, it has been shown that naturally occurring polysulfides and ITCs (e.g., sulforaphane, benzylisothiocianate) act on cells and tissues through H_2_S as a mediator [39]. While whether GTL, GER, and *L. sativum* seed extract could release H_2_S is still unknown, *E. sativa* seed extract has been identified to display an L-cysteine-independent H_2_S donation [39]. Interestingly, we previously showed that the low-mineralizing behavior of hMSCs correlated with lower expression of the H_2_S endogenous biosynthetic pathway in MSCs [42]. Consequently, exogenous H_2_S may compensate, at least for *E. sativa* seed extract, for the lower constitutive expression in low-mineralizing MSCs.

Overall, this in vitro preclinical study aimed at answering the knowledge gap of whether *Brassica* seed extracts comprise the same biological properties as their major GLSs. Here, we confirmed that *Brassica* seed extracts and their major GLSs constituents showed a similar ability to induce osteogenic differentiation in hMSCs. Moreover, this study expands the knowledge on the possible application of cruciferous derivatives as natural alternatives for the prevention and treatment of bone-deteriorating pathologies. However, whether the use of whole extracts or purified GLSs would be preferable is still an open question. Further in vitro/in vivo preclinical studies and clinical studies need to be performed to specifically address the potential protective role of OSCs against bone loss compared to the crude *Brassica* seed extract by including a controlled measurement of GLSs and their by-products in the circulation and tissue and a correlation to H_2_S blood levels and bone status to define preventive/clinical dietary protocols for patients with an increased risk of bone fragility.

## 5. Conclusions

This study showed that OSCs belonging to the class of GLSs play an anabolic role on hMSCs. Most importantly, by providing a direct comparison of the effect of *Brassica* seed extracts and their most representative GLSs on hMSCs, they support the hypothesis that they constitute a likely biological mechanism for the improved skeletal health observed in patients who maintain an elevated intake of cruciferous vegetables. Direct comparisons on extracts showed that *E. sativa* induced a higher level of osteogenesis compared to the most published species of *Brassica* extract, *L. sativum*. Regarding GLSs, we found that GTL induces higher osteogenesis than GER. However, these differences in the stimulatory effect of different compounds are not extremely marked, thus evidencing an overall capacity of osteogenic stimulation of *Brassica* extracts and GLSs. Further preclinical in vivo studies and clinical studies need to be performed to specifically address the potential protective role of OSCs derived from *Brassicaceae* against bone loss compared to whole-seed extracts.

## Figures and Tables

**Table 1:** List of primer sequences. FW: forward primer; REV: reverse primer.

Gene	Protein		5′-Sequence-3′	Product Size (bp)	Accession Number
GAPDH	Glyceraldehyde-3 phosphate dehydrogenase	FW	CGGAGTCAACGGATTTGG	218	NM_002046
		REV	CCTGGAAGATGGTGATGG		
ALP	Alkaline phosphatase	FW	GGAAGACACTCTGACCGT	152	NM_000478
		REV	GCC CAT TGC CAT ACA GGA		
BSP	Bone sialoprotein	FW	CAGTAGTGACTCATCCGAAG	158	NM_004967
		REV	CATAGCCCAGTGTTGTAGCA		
SMAD1	SMAD Family Member 1	FW	CACCCGTTTCCTCACTCTCC	257	NM_005900
		REV	TCCTCATAAGCAACCGCCTG		
WISP1	WNT1-inducible-signaling pathway protein 1	FW	ACACGCTCCTATCAACCCAAG	103	NM_003882
		REV	CATCAGGACACTGGAAGGACA		

**Table 2:** P-values of two-way ANOVA. ES (*Eruca sativa*), LS (*Lepidium sativum*), GER (glucoerucin), GTL (benzylglucosinolate/glucotropaeolin); D (day); ns = non significant.

Results of Two-Way ANOVA	Influence of Concentration (D14)	Influence of Concentration (D21)	Influence of Donor (D14)	Influence of Donor (D21)
ES	*p* < 0.01	ns	*p* < 0.0001	*p* < 0.0001
GER	*p* < 0.0001	ns	*p* < 0.0001	*p* < 0.0001
LS	*p* < 0.01	ns	*p* < 0.0001	*p* < 0.0001
GTL	*p* < 0.001	ns	*p* < 0.0001	*p* < 0.0001

## Data Availability

Data for this article, including raw data used for statistical analyses, are available at FIGSHARE at https://doi.org/10.6084/m9.figshare.c.7453087.v1 (accessed on 1 September 2024).

## Supplementary Materials

The following supporting information can be downloaded at: https://www.mdpi.com/article/10.3390/nu16203457/s1, Figure S1: mRNA expression of SMAD-1 in GTL-treated cells vs. CTRL cells during osteogenic stimulation at D14.

## References

[1] Beetch M., Harandi-Zadeh S., Shen K., Lubecka K., Kitts D.D., O’Hagan H.M., Stefanska B. (2020). Dietary antioxidants remodel DNA methylation patterns in chronic disease. Br. J. Pharmacol..

[2] Gambari L., Cellamare A., Grassi F., Grigolo B., Panciera A., Ruffilli A., Faldini C., Desando G. (2022). Overview of Anti-Inflammatory and Anti-Nociceptive Effects of Polyphenols to Halt Osteoarthritis: From Preclinical Studies to New Clinical Insights. Int. J. Mol. Sci..

[3] Houghton C.A. (2019). Sulforaphane: Its “Coming of Age” as a Clinically Relevant Nutraceutical in the Prevention and Treatment of Chronic Disease. Oxid. Med. Cell Longev..

[4] Lopez-Otin C., Kroemer G. (2021). Hallmarks of Health. Cell.

[5] de Sire A., de Sire R., Curci C., Castiglione F., Wahli W. (2022). Role of Dietary Supplements and Probiotics in Modulating Microbiota and Bone Health: The Gut-Bone Axis. Cells.

[6] Lambert M.N.T., Jeppesen P.B. (2018). Isoflavones and bone health in perimenopausal and postmenopausal women. Curr. Opin. Clin. Nutr. Metab. Care.

[7] Rizzoli R., Biver E., Brennan-Speranza T.C. (2021). Nutritional intake and bone health. Lancet Diabetes Endocrinol..

[8] Sharma A.R., Lee Y.H., Bat-Ulzii A., Chatterjee S., Bhattacharya M., Chakraborty C., Lee S.S. (2023). Bioactivity, Molecular Mechanism, and Targeted Delivery of Flavonoids for Bone Loss. Nutrients.

[9] Benetou V., Orfanos P., Feskanich D., Michaelsson K., Pettersson-Kymmer U., Byberg L., Eriksson S., Grodstein F., Wolk A., Jankovic N. (2018). Mediterranean diet and hip fracture incidence among older adults: The CHANCES project. Osteoporos. Int..

[10] Benetou V., Orfanos P., Pettersson-Kymmer U., Bergstrom U., Svensson O., Johansson I., Berrino F., Tumino R., Borch K.B., Lund E. (2013). Mediterranean diet and incidence of hip fractures in a European cohort. Osteoporos. Int..

[11] Blekkenhorst L.C., Hodgson J.M., Lewis J.R., Devine A., Woodman R.J., Lim W.H., Wong G., Zhu K., Bondonno C.P., Ward N.C. (2017). Vegetable and Fruit Intake and Fracture-Related Hospitalisations: A Prospective Study of Older Women. Nutrients.

[12] Munoz-Garach A., Garcia-Fontana B., Munoz-Torres M. (2020). Nutrients and Dietary Patterns Related to Osteoporosis. Nutrients.

[13] Rizzoli R. (2022). Dairy products and bone health. Aging Clin. Exp. Res..

[14] Lu Y., Zhang M., Huang D. (2022). Dietary Organosulfur-Containing Compounds and Their Health-Promotion Mechanisms. Annu. Rev. Food Sci. Technol..

[15] Ruhee R.T., Roberts L.A., Ma S., Suzuki K. (2020). Organosulfur Compounds: A Review of Their Anti-inflammatory Effects in Human Health. Front. Nutr..

[16] Gambari L., Grigolo B., Grassi F. (2022). Dietary organosulfur compounds: Emerging players in the regulation of bone homeostasis by plant-derived molecules. Front. Endocrinol..

[17] Đulović A., Koch M.A., Thongyoo P., Pattison D.I., Blažević I., Rollin P., Agerbirk N. (2024). Glucosinolates in non-Brassicales plant species: Critical literature evaluation and testing of two high chemical quality reports. Biochem. Syst. Ecol..

[18] Bhat R., Vyas D. (2019). Myrosinase: Insights on structural, catalytic, regulatory, and environmental interactions. Crit. Rev. Biotechnol..

[19] Cebeci F., Mayer M.J., Rossiter J.T., Mithen R., Narbad A. (2022). Molecular Cloning, Expression and Characterisation of a Bacterial Myrosinase from Citrobacter Wye1. Protein J..

[20] Francis F., Lognay G., Wathelet J.P., Haubruge E. (2002). Characterisation of aphid myrosinase and degradation studies of glucosinolates. Arch. Insect Biochem. Physiol..

[21] Blazevic I., Montaut S., Burcul F., Olsen C.E., Burow M., Rollin P., Agerbirk N. (2020). Glucosinolate structural diversity, identification, chemical synthesis and metabolism in plants. Phytochemistry.

[22] Prieto M.A., Lopez C.J., Simal-Gandara J. (2019). Glucosinolates: Molecular structure, breakdown, genetic, bioavailability, properties and healthy and adverse effects. Adv. Food Nutr. Res..

[23] Abdull Razis A.F., Bagatta M., De Nicola G.R., Iori R., Ioannides C. (2011). Up-regulation of cytochrome P450 and phase II enzyme systems in rat precision-cut rat lung slices by the intact glucosinolates, glucoraphanin and glucoerucin. Lung Cancer.

[24] Baird W.M., Zennie T.M., Ferin M., Chae Y.H., Hatchell J., Cassady J.M. (1988). Glucolimnanthin, a plant glucosinolate, increases the metabolism and DNA binding of benzo[a]pyrene in hamster embryo cell cultures. Carcinogenesis.

[25] Schlotz N., Odongo G.A., Herz C., Wassmer H., Kuhn C., Hanschen F.S., Neugart S., Binder N., Ngwene B., Schreiner M. (2018). Are Raw Brassica Vegetables Healthier Than Cooked Ones? A Randomized, Controlled Crossover Intervention Trial on the Health-Promoting Potential of Ethiopian Kale. Nutrients.

[26] Gambari L., Barone M., Amore E., Grigolo B., Filardo G., Iori R., Citi V., Calderone V., Grassi F. (2022). Glucoraphanin Increases Intracellular Hydrogen Sulfide (H_2_S) Levels and Stimulates Osteogenic Differentiation in Human Mesenchymal Stromal Cell. Nutrients.

[27] Calvey E.M., White K.D., Matusik J.E., Sha D., Block E. (1998). Allium chemistry: Identification of organosulfur compounds in ramp (Allium tricoccum) homogenates. Phytochemistry.

[28] Sim M., Blekkenhorst L.C., Lewis J.R., Bondonno C.P., Devine A., Zhu K., Woodman R.J., Prince R.L., Hodgson J.M. (2018). Vegetable Diversity, Injurious Falls, and Fracture Risk in Older Women: A Prospective Cohort Study. Nutrients.

[29] Sim M., Blekkenhorst L.C., Lewis J.R., Bondonno C.P., Devine A., Zhu K., Woodman R.J., Prince R.L., Hodgson J.M. (2018). Vegetable and fruit intake and injurious falls risk in older women: A prospective cohort study. Br. J. Nutr..

[30] Atanasov A.G., Waltenberger B., Pferschy-Wenzig E.M., Linder T., Wawrosch C., Uhrin P., Temml V., Wang L., Schwaiger S., Heiss E.H. (2015). Discovery and resupply of pharmacologically active plant-derived natural products: A review. Biotechnol. Adv..

[31] Abdallah H.M., Farag M.A., Algandaby M.M., Nasrullah M.Z., Abdel-Naim A.B., Eid B.G., Safo M.K., Koshak A.E., Malebari A.M. (2020). Osteoprotective Activity and Metabolite Fingerprint via UPLC/MS and GC/MS of Lepidium sativum in Ovariectomized Rats. Nutrients.

[32] Dixit V., Kumar I., Palandurkar K., Giri R., Giri K. (2020). Lepidium sativum: Bone healer in traditional medicine, an experimental validation study in rats. J. Family Med. Prim. Care.

[33] El-Haroun H. (2020). Comparative Study on the Possible Protective Effect of Lepidium Sativum versus Teriparatide in Induced Osteoporosis in Adult Male Guinea Pigs. Egiptian J. Hystol..

[34] Elshal M.F., Almalki A.L., Hussein H.K., Khan J.A. (2013). Synergistic antiosteoporotic effect of Lepidium sativum and alendronate in glucocorticoid-induced osteoporosis in Wistar rats. Afr. J. Tradit. Complement. Altern. Med..

[35] Jeong J., Park H., Hyun H., Kim J., Kim H., Oh H.I., Hwang H.S., Kim D.K., Kim H.H. (2015). Effects of Glucosinolates from Turnip (*Brassica rapa* L.) Root on Bone Formation by Human Osteoblast-Like MG-63 Cells and in Normal Young Rats. Phytother. Res..

[36] Juma A. (2007). The effects of Lepidium sativum seeds on fracture-induced healing in rabbits. MedGenMed.

[37] Lazzeri L., Malaguti L., Cinti S., Ugolini L., De Nicola G.R., Bagatta M., Casadei N., D’Avino L., Matteo R., Patalano G. (2013). The Biofumigation System for Plant Cultivation and Defence. An Italian Twenty-Year Experience of Study and Application. Acta Hortic..

[38] Lucarini E., Pagnotta E., Micheli L., Parisio C., Testai L., Martelli A., Calderone V., Matteo R., Lazzeri L., Di Cesare Mannelli L. (2019). Eruca sativa Meal against Diabetic Neuropathic Pain: An H2S-Mediated Effect of Glucoerucin. Molecules.

[39] Testai L., Pagnotta E., Piragine E., Flori L., Citi V., Martelli A., Mannelli L.D.C., Ghelardini C., Matteo R., Suriano S. (2022). Cardiovascular benefits of Eruca sativa mill. Defatted seed meal extract: Potential role of hydrogen sulfide. Phytother. Res..

[40] Wathelet J.P., Iori R., Leoni O., Quinsac A., Palmieri S. (2004). Quinsac Guidelines for glucosinolate analysis in green tissues used for biofumigation. Agroindustria.

[41] Flori L., Montanaro R., Pagnotta E., Ugolini L., Righetti L., Martelli A., Mannelli L.D., Ghelardini C., Brancaleone V., Testai L. (2023). Erucin Exerts Cardioprotective Effects on Ischemia/Reperfusion Injury through the Modulation of mitoKATP Channels. Biomedicines.

[42] Gambari L., Lisignoli G., Gabusi E., Manferdini C., Paolella F., Piacentini A., Grassi F. (2017). Distinctive expression pattern of cystathionine-beta-synthase and cystathionine-gamma-lyase identifies mesenchymal stromal cells transition to mineralizing osteoblasts. J. Cell Physiol..

[43] Gambari L., Grigolo B., Filardo G., Grassi F. (2020). Sulfurous thermal waters stimulate the osteogenic differentiation of human mesenchymal stromal cells—An in vitro study. Biomed. Pharmacother..

[44] Zou M.L., Chen Z.H., Teng Y.Y., Liu S.Y., Jia Y., Zhang K.W., Sun Z.L., Wu J.J., Yuan Z.D., Feng Y. (2021). The Smad Dependent TGF-beta and BMP Signaling Pathway in Bone Remodeling and Therapies. Front. Mol. Biosci..

[45] French D.M., Kaul R.J., D’Souza A.L., Crowley C.W., Bao M., Frantz G.D., Filvaroff E.H., Desnoyers L. (2004). WISP-1 is an osteoblastic regulator expressed during skeletal development and fracture repair. Am. J. Pathol..

[46] Gordon J.A., Tye C.E., Sampaio A.V., Underhill T.M., Hunter G.K., Goldberg H.A. (2007). Bone sialoprotein expression enhances osteoblast differentiation and matrix mineralization in vitro. Bone.

[47] Vimalraj S. (2020). Alkaline phosphatase: Structure, expression and its function in bone mineralization. Gene.

[48] Lucarini E., Micheli L., Pagnotta E., Matteo R., Parisio C., Toti A., Ferrara V., Ciampi C., Martelli A., Testai L. (2022). Beneficial Effects of Eruca sativa Defatted Seed Meal on Visceral Pain and Intestinal Damage Resulting from Colitis in Rats. Foods.

[49] Sreeja P.S., Arunachalam K., Martins D.T.O., Lima J., Balogun S.O., Pavan E., Saikumar S., Dhivya S., Kasipandi M., Parimelazhagan T. (2018). Sphenodesme involucrata var. paniculata (C.B. Clarke) Munir.: Chemical characterization, anti-nociceptive and anti-inflammatory activities of methanol extract of leaves. J. Ethnopharmacol..

[50] Choi H., Kim H., Han S., Park H.W., Ha I.J., Kim J.S., Lee S.G. (2023). Antioxidant and Anti-Inflammatory Activities of High-Glucosinolate-Synthesis Lines of Brassica rapa. Antioxidants.

[51] Grassi F., Tyagi A.M., Calvert J.W., Gambari L., Walker L.D., Yu M., Robinson J., Li J.Y., Lisignoli G., Vaccaro C. (2016). Hydrogen Sulfide Is a Novel Regulator of Bone Formation Implicated in the Bone Loss Induced by Estrogen Deficiency. J. Bone Miner. Res..

