# Supplementary material for: Insights into Osteogenesis Induced by Crude Brassicaceae Seeds Extracts: A Role for Glucosinolates
Source: Nutrients. 2024 Oct 12;16(20):3457. doi: 10.3390/nu16203457 (PMC11510261; doi:10.3390/nu16203457)
Supplement: Supplementary file 1 [file nutrients-16-03457-s001.zip › nutrients-3252293-supplementary.pdf]

Supplementary information

1. **mRNA expression of SMAD-1 in GTL -treated cells vs CTRL cells during osteogenic stimulation at D14**

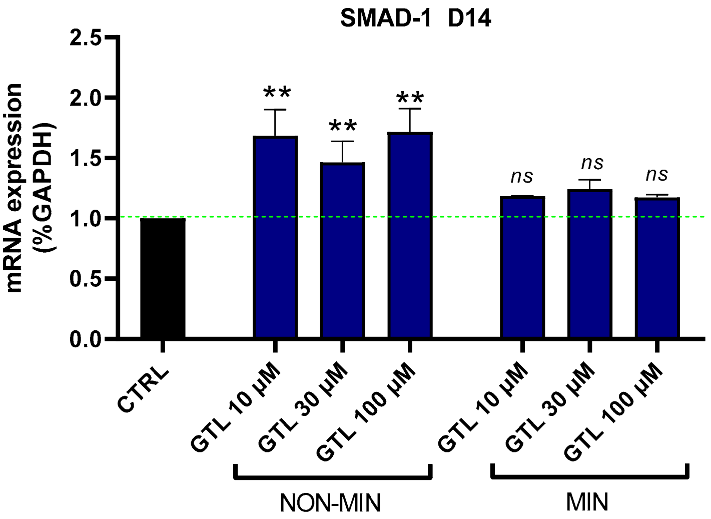

Supplementary Figure 1: mRNA expression of SMAD-1 in GTL -treated cells vs CTRL cells during osteogenic stimulation at D14. Histogram shows mean ± sem of mRNA expression in duplicates (fold increase) of non-mineralizing donors *vs* mineralizing donors. The Wilcoxon Signed Rank Test was performed. ** $p<0.01$.
